# Staff perspectives on a novel pediatric developmental screening and referral program in a community health center

**DOI:** 10.3389/fped.2026.1872417

**Published:** 2026-09-04

**Authors:** Jessica McCann, MacKenzie Koester, Rebecca L. Emery Tavernier, Alexandra Munro, April Joy Damian

**Affiliations:** 1 Moses/Weitzman Health System, Middletown, CT, United States; 2 Department of Family Medicine and Biobehavioral Health, University of Minnesota Medical School, Duluth, MN, United States

**Keywords:** community health center, developmental screening, early childhood development, implementation, safety net

## Abstract

There are longstanding gaps in the surveillance, screening, and referral for developmental disorders among medically underserved children, which exacerbate existing health inequities. While innovative interventions to close these gaps are emerging, little is known about clinician and staff perspectives on implementing these novel screening and referral approaches for developmental disorders within safety net settings. This study examines safety net clinician and staff perspectives on implementing the Early Childhood Initiative, a project aimed at detecting developmental concerns early and facilitating timely intervention for children up to age five years within a large community health center-based pediatric practice in Connecticut. Initiative staff created clinic workflows within electronic health records to implement and track referral pathways, including outreach, education, and community connections, screening 8,095 children between September 2023 and August 2025. To better understand program implementation, 14 semi-structured interviews were conducted with the project team using the Reach, Effectiveness, Adoption, Implementation, and Maintenance (RE-AIM) framework. Overall, the project team was enthusiastic about the effectiveness of the Early Childhood Initiative but had concerns related to gaps in families served (Reach), training discrepancies (Implementation), staff turnover (Adoption), and sustainability (Maintenance). The Early Childhood Initiative offers a scalable strategy for early detection of and intervention for developmental disorders in safety net settings, potentially reaching families often left out of the process. However, implementation challenges suggest a need for further refinement.

## 1 Introduction

Developmental screening and surveillance are important public health tools for supporting developmental trajectories. The American Academy of Pediatrics (AAP) recommends screening for developmental and behavioral disorders at regular intervals during well-child visits in the first 30 months of life using validated tools (1). In addition, developmental surveillance should occur at every visit by leveraging caregiver input and clinical observation (2). Regular screening and surveillance enables timely identification of developmental concerns and expedites referral to appropriate interventions, which improves long-term outcomes and results in significant cost savings over the life course (3, 4).

Although pediatrician use of developmental screening tools increased 174% between 2002 and 2016, gaps persist (5). The most recent data from the National Survey of Children's Health show that only 33% of children received developmental surveillance from a health care provider and 36% had a parent complete a developmental screening tool in the previous year (6). Barriers to receiving recommended services among children with identified developmental concerns disproportionately affect historically marginalized populations (7). For example, racially or ethnically minoritized children are less likely than other groups to receive timely developmental screening or specialized services, as are low-income and publicly-insured children (8–10). Despite growing availability of Spanish-language screening tools, families with limited English proficiency have lower odds of receiving screening or surveillance than English speaking families (11, 12). Children of parents with low health literacy also experience barriers to accessing the resources needed to facilitate screening and early intervention referral (13). Importantly, the same subgroups of children who experience gaps in screening, surveillance, and intervention for developmental disorders are at heightened risk for developmental disorders due to a complex array of social, environmental, and systemic factors, making it especially critical they receive timely and appropriate care (1, 14–16).

Several interventions have attempted to address disparities in developmental screening and surveillance using nontraditional settings or technological efficiencies. For example, administering developmental screening interventions within Special Supplemental Nutrition Program for Women, Infants, and Children (WIC) clinics (17) and child care settings (18) can facilitate screening opportunities for low income families, as can leveraging community health workers (CHWs) as health educators and family supports (19). Adjusting screeners to incorporate visually-based tools (i.e., images with minimal text) can effectively increase screening rates for autism spectrum disorder among low-literacy or non-English speaking populations (20). Transitioning to digital screening and/or digital workflows can also successfully increase screening rates among vulnerable populations while promoting cost savings and reducing errors (21–24). Although these interventions show promise in promoting developmental screening uptake, there is a need to further understand barriers to enhancing developmental screening and referral practices. This is especially true in safety net settings like community health centers, which provide care regardless of a patient's ability to pay. Typically, safety net settings operate on tight margins, with limited funding from federal, state, and local governments, creating resource and staffing constraints that can inhibit developmental screening program implementation (25).

Staff and clinician perspectives on developmental screening tools, workflows, and referral practices are limited (9, 25), particularly among teams working in safety net settings (16) and those implementing culturally relevant developmental screening practices (26). This study seeks to address this limitation by using the Reach, Effectiveness, Adoption, Implementation, and Maintenance (RE-AIM) framework (27) to understand staff and clinician experiences implementing a novel, culturally relevant initiative to increase developmental screening and referral in a community health center. The RE-AIM framework was selected because it is widely used in implementation science and provides a sound structure for understanding implementation outcomes. Moreover, because RE-AIM was initially developed to evaluate program scalability in community and clinical settings (28), it was determined to be particularly relevant for the aims of the current study.

## 2 Methods

### 2.1 Early childhood initiative

The Early Childhood Initiative (ECI) was funded by the Health Resources & Services Administration (H8KCS49677; Munro, PI) and implemented between September 2023 and August 2025 within Community Health Center, Inc. (CHCI), a federally supported community health center in Connecticut. CHCI patients are predominately low income and most identify as a racial or ethnic minority (29). The primary aims of ECI were to (1) increase the number of children aged zero to five years who receive all AAP-recommended developmental screenings and (2) increase the number of families who receive follow-up and appropriate services within 30 days of a positive developmental screen. ECI focused on staffing and technological innovations to facilitate screening and referral tracking.

As part of CHCI's typical screening and referral workflow, developmental screening and surveillance is completed during well-child visits following AAP-recommended schedules and validated tools, including the Modified Checklist for Autism for Toddlers and the Parents' Evaluation of Developmental Status for children younger than three years and the Pediatric Symptom Checklist for children aged three to five years (30). If a primary care provider identifies an area of concern for a child younger than three years during a well-child visit, the family is referred to Birth to Three (B23) services, a statewide program in Connecticut offering referrals and supports for children with developmental disabilities and their families. When a developmental concern is identified for children aged three to five years, the family is connected to local school-based services.

ECI enhanced this standard screening and referral workflow in several ways. First, ECI integrated a dedicated care coordinator and a CHW, who personally followed up with families within 30 days of a positive developmental screen, offering education or other services in English or Spanish to facilitate these connections. Next, while CHCI has historically worked closely with B23 and school-based staff to serve families, ECI further enabled the addition of a subject matter expert from the state Office of Early Childhood, who facilitated new connections and provided insights into program design and referral processes. This state partner closely collaborated with the care coordinator and CHW to troubleshoot when necessary and ensure families received the most appropriate referrals. Third, while clinical providers or medical assistants previously documented screening and referral-related clinical touchpoints within the electronic health record (EHR), ECI standardized these steps by leveraging CHCI's business intelligence, information technology, and population health teams to deploy a series of EHR workflows. These workflows were outlined in process maps and flow diagrams and summarized in “playbooks,” which detailed each step in the screening, referral, and follow-up process and the staff and technology involved in each step. The process map for children under age three (Supplementary Figure S1) included provider-initiated screening, consenting, referrals to B23 services, and follow-ups; community health worker and care coordinator engagements with families; missed appointment triggers for population health staff; and additional documentation by medical assistants and business intelligence teams. The process map for children aged three to five (Supplementary Figure S2) included these steps as well as detailed processes for school-based service referrals, requests for school district-issued records like Individualized Education Plans, and service referral options for children deemed ineligible by school districts. Additionally, ECI built on CHCI's existing text messaging platform to engage families using appointment reminders and links to online tools like the AAP's Child Health Tracker. Finally, ECI included the deployment of internal data dashboards enabling program staff to easily visualize the status of screenings, referrals, and longer-term outcomes for each patient with identified concerns. These dashboards, as seen in Supplementary Figure S3, include visit data (date, provider, CHCI site, diagnosis) and screening and referral status, allowing the program coordinator and CHW to quantify, prioritize, and tailor outreach and follow-up. CHCI staff received role-appropriate training, including a grand rounds-style session for clinicians, though formal training was not offered for external staff.

### 2.2 Ethical considerations

The CHCI Institutional Review Board (IRB) classified this study as exempt.

### 2.3 Recruitment

CHCI staff who were involved with program implementation were invited to participate in interviews. Eligible staff included those who assisted with grant writing and technological processes, medical and behavioral health providers, and the ECI care coordinator and CHW. Recruitment and interviews occurred concurrently during June and July of 2025.

### 2.4 Interview guide

The team used the RE-AIM framework to create a semi-structured interview guide designed to assess specific areas of ECI implementation (Supplementary Table S4) (27). Questions were designed to elicit feedback and allow for respondent and interviewer flexibility. Nine questions, with additional probes, assessed the RE-AIM dimensions (31).

### 2.5 Interview procedures

Three trained researchers with no relationship to interviewees nor any affiliation with the ECI program led the interviews. The interviews took place virtually via Zoom, were recorded with participant verbal consent, were transcribed verbatim utilizing Zoom transcription software, and were reviewed for accuracy (32). Interview questions were written for 30-min timeslots to accommodate busy staff schedules. Interviewees received no incentives for participation.

### 2.6 Qualitative analysis strategy

As has been done in prior qualitative research guided by the RE-AIM framework, researchers used a framework analysis approach, a type of qualitative content analysis well-suited for studies guided by *a priori* conceptual frameworks (33). In this study, RE-AIM dimensions were used as deductive codes. Two researchers trained in qualitative analysis independently familiarized themselves with the transcripts, then indexed interview responses deductively based on RE-AIM dimensions. The coding of quotes into RE-AIM dimensions was iteratively reviewed and refined through discussion until consensus was reached. Data were then charted in a final analytic framework that included definitions of the RE-AIM dimensions (31) and illustrative quotes (Supplementary Table S5). Researchers then identified patterns within this matrix, which were summarized across participants. Final interpretation was conducted by examining relationships within and across RE-AIM dimensions. Due to the small number of interviews, researchers performed all analyses using tabular functions within Microsoft Word (34).

## 3 Results

### 3.1 Participant characteristics

Twenty program staff were invited to interview, and 14 interviews were conducted. Among the interviewed program staff, 50% (*n* = 7) were medical or behavioral health providers, 29% (*n* = 4) were in leadership positions (i.e., grant champions, stakeholders, and clinical directors), 14% (*n* = 2) were implementation staff (i.e., the care coordinator and CHW), and 1% (*n* = 1) supported the project as a business intelligence developer. Sixty-four percent (*n* = 9) of interviewees were female and 36% (*n* = 5) were male. Among the six staff who did not participate, five were medical providers and one assisted with program design as a quality improvement coach.

### 3.2 Analysis results

Patterns related to each of the five RE-AIM dimensions were documented (see Table 1 for additional exemplary quotes by dimension).

**Table 1:** Quotes from clinicians, leadership, implementation staff, and support staff, organized by RE-AIM dimension, with definitions adapted from Holtrop et al. (31).

Re-aim dimension	Definition	Quote and respondent type
Reach	Were target families served by the ECI program, were gaps recognized, and, if so, how could these gaps be addressed?	“Some families don't think their kids need any sort of interventions…they might grow out of it.”—Clinician “It sometimes amazes me, how much parents are concerned about their children's development…particularly early in life, and how distracted they are away from that…by things like safety, adequate housing, a multitude of things.”—Clinician “The three-to-five age range is the harder target range, because once you finish [the Connecticut] Birth to Three [program], it's not as cut and dry getting services.”—Clinician “Because we don't offer the evaluations in Spanish…that would be really helpful.”—Clinician “I think concerns about [the Department of Children and Families] and immigration play into some decisions to refuse.”—Leadership “We do the best we have as far as a direct translation from the English…but the way that the questions are phrased…may not cross over from the United States-focused approach.”—Leadership
Effectiveness	Did the program successfully achieve its intended outcomes of early childhood screening and closed-loop follow-ups, and what barriers or facilitators influenced this?	“When we're working together…we were able to…get more families to services and supports.”—*Implementation Staff* “We're seeing more families accept services and get the support that their child and family may need.”—*Implementation Staff* “I'm not as…fully well-versed in what is part of the entire program, other than having what we have…at the site right now.”—*Clinician*
Adoption	Did the organization successfully apply all aspects of the intervention, which barriers and facilitators influenced program adoption, and were differences observed by role?	“It makes you work harder to make sure these families have what they need.”—*Implementation Staff* “There is absolutely…leadership buy in.”—*Leadership*
Implementation	Was CHCI able to successfully implement the ECI program, what factors made implementation easier or more difficult, and which adaptations were necessary?	“[The training] was absolutely adequate…I had the in-person training which was a little over a month.”—*Implementation Staff* “[Data] sometimes felt like a barrier, but I think everyone tried to do their best to work together.”—*Support Staff* “The three-to-five [age range] process…was…brand new…so folks definitely needed to adapt…I think that can be very challenging.”—*Clinician* “Every single district has a separate process.”—Implementation *Staff* “Once kids hit three and transition to the [school system]…we still struggle with that workflow.”—*Leadership*
Maintenance	What internal or external factors may affect long-term program sustainability or what may need to be modified to ensure this?	“Use evolving and emerging and developed technology to bypass some of the problems that create that added effort and labor.”—*Clinician*

#### 3.2.1 Reach

Over the course of the two-year program, 8,983 well-child visits occurred, with 90% (*n* = 8,095) receiving developmental screening. Of those screened, clinicians identified developmental concerns and made appropriate referrals among 1,274 children (16%), following up with 937 children (74%) within 30 days of referral. Interviewees across roles noted that the program provided the documentation and context needed to follow-up and assess future developmental evaluation, referral, and service readiness. However, leadership, clinical, and implementation staff also identified gaps in reach. Staff reported that some families declined evaluations and referrals, citing cultural stigma, belief that their child would “grow out of it,” fear of government intervention (i.e., immigration, children's services), and the need to prioritize housing and other basic necessities over healthcare. Other families were lost to follow-up for unknown reasons. Families with children between the ages of three and five were especially likely to be lost to follow-up due to the workflow and referral process for this age range, which involved handoffs to school systems with fragmented policies and procedures. Moreover, both clinicians and leadership noted a gap in program reach for families with limited English proficiency. Staff noted these gaps typically originated during the initial screening process and may stem from the “United States-focused” phrasing used in the screener. Accordingly, one member of the leadership team stated:

“My hypothesis would be that…we disproportionately miss early diagnosis in non-English speaking patients.”

#### 3.2.2 Effectiveness

When families were successfully reached, all interviewees found that ECI was effective for meeting the needs of the children and families they served. Staff across roles recognized the need for developmental screening and referrals and found that ECI allowed for more timely delivery of these services and connection to follow-up, with one of the implementation staff noting:

“We're seeing more families accept services and get the support that their child and family may need.”

However, both clinical and implementation staff also commonly noted that gaps in provider knowledge may have reduced program effectiveness. Interviewees stated that, while all staff understood essential components, the program's many moving parts (e.g., multiple CHCI sites, referral pathways and partners, staff touchpoints) and complicated flowcharts made it difficult for medical providers to easily grasp all ECI procedures and offerings. One provider stated:

“It was a lot of information, and I think we had a hard time… grappling with that flowchart.”

#### 3.2.3 Adoption

Although program staff were highly motivated to adopt the program and recognized its benefits, they also mentioned barriers that impacted program adoption. Interviewees noted a lack of clinician response to needed program adaptations, such as changes to the referral process for children in the three-to-five age range, which likely hindered adoption among overwhelmed clinical staff. For example, one clinician noted:

“I remember that they were talking about the way to refer the three-to-five age range. I never managed to integrate that fully into my clinical practice, because I found it very cumbersome and difficult.”

Both clinical and non-clinical staff also perceived that some medical providers were resistant to adopting new workflows and would benefit from more support in implementing such changes. For example, one implementation staff member noted:

“That’s been the trickiest—just making sure…that all the providers know that we have our process, and that there’s a process that they could follow that we've implemented to make their lives easier. So, I think a lot of providers…have their own methods that have worked for them.”

Adoption was further impacted by both staff and leadership turnover, which also likely reduced program effectiveness. Despite the consensus that senior leadership was invested in the program, leadership changes led to disruptions in adoption, while provider turnover impacted buy-in, leading to discontinuity in care and inconsistent program uptake. These observations were best summarized by a clinician and a member of the leadership team, respectively:

“After a change in leadership…things fell through the cracks…”

“Anytime you have turnover…in the provider team…that can create discontinuity for the patient care and the well-child screening process.”

#### 3.2.4 Implementation

Notably, all program staff stated that the ECI was appropriate for their professional role and duties. Interviewees across roles also commonly noted that staff dedication and collaboration led to program successes, even during challenging aspects of implementation. Specifically, clinical staff repeatedly mentioned the importance of the care coordinator and CHW, who made themselves available to families and program staff alike, with one clinician stating:

“Having [program staff] there for me to go up to them and ask them questions…That has made a huge difference.”

Program staff also described how the data dashboards allowed them to easily view and track referrals and follow-up status across multiple CHCI sites, which also directly improved program reach. One implementation staff member stated:

“A dashboard was programmed to reel in all the referrals…It’s been great.”

However, some team members identified disparities in training processes as a primary barrier to program implementation. While some staff felt their training was adequate and felt confident in their roles and duties, others felt the provider training was confusing and additional check-ins, especially reminders on programmatic offerings, would have been helpful. Staff specifically mentioned the complexity of both ECI processes and the flowcharts (Supplementary Figures S1 and S2), which also impacted program effectiveness. One health center leader noted:

“I think [training] overwhelmed the [medical] staff. They came out just sort of mystified.”

To further support implementation, program staff mentioned making small adaptations to support the program, such as adjusting the referral process for the three-to-five age range and altering processes for collaborating with local school districts. Despite program implementation staff noting that evaluations and services might be underutilized with this population because workflows are more complicated than those pertaining to younger children, staff adoption of these changes varied. Additionally, improvements in standardizing communication with school districts are essential as the current process is cumbersome and inefficient, as acknowledged by one implementation staff member:

“We had to make changes…taking the work off providers and taking on more responsibility in the hopes that this would allow providers to refer more children within the three-to-five range.”

#### 3.2.5 Maintenance

While interviews revealed facilitators discussed above that support program success and sustainability, staff commonly identified limited staffing, specifically among clinicians, care coordinators, and CHWs, as a potential threat to sustainability. Program staff viewed the latter two roles as essential to program success and felt that additional staff in these roles would promote sustainability. Additionally, several interviewees cited lack of funding as a potential threat or barrier to program continuation, as noted by one implementation staff member:

“It always comes down to funding, but…we're doing a great job now.”

Finally, support staff, clinicians, and implementation staff expressed a desire to optimize available technology to assist medical providers with the screening and referral processes, allowing for optimal data tracking, accessible outcome monitoring, and reduced provider burden.

## 4 Discussion

This work documents clinician and program staff perceptions of a novel developmental screening and referral process in a community health center. Overall, team members were satisfied with the program and found it to effectively increase developmental screening and closed-loop referrals. However, many noted a need for program refinement to address issues related to program reach, adoption, implementation, and maintenance. In particular, staff noted that changes were needed to support uptake and understanding of the program, with others noting concerns for program sustainability.

Across RE-AIM dimensions, the ECI program was viewed as both helpful and important, contributing to shared buy-in among staff and leadership and facilitating its adoption into the clinical workflow. Factors like open communication and a strong sense of team collaboration facilitated program implementation and adoption as did program adaptations that supported feasibility. For example, adjustments to referral workflows for children aged three to five years allowed staff to uphold the core components of the program while making changes to support its effectiveness for particular age groups. These types of minor adjustments are commonly accepted in similar public health interventions (22, 35) and illustrate the importance of program flexibility. Despite these successes, barriers to the program were identified. Staff commonly noted that programmatic enhancements in language diversity, particularly in the screening phase, would help address disparities in reach. This is consistent with literature noting language-based disparities in primary care screening, particularly among non-English or Spanish speakers (11, 12). As 35% of families served by CHCI report limited English proficiency (29), leveraging linguistically and culturally concordant staff and screening tools, rather than relying on literal translations and interpreters, could improve reach, as noted in other health center-based studies (36). Reaching families who declined services presented another challenge, with staff noting that a deeper understanding of each family's lived experience would allow a more tailored approach to address concerns like stigma. Findings indicating increased needs for culturally-relevant services are not unique to this study; 30% of all U.S. immigrants identify health centers as their usual source of care (37) and prior work points to the importance of integrating comprehensive approaches to address diverse patient perspectives (38). Relatedly, one member of the leadership team mentioned that strengthening partnerships with community organizations could begin to address these and other issues, potentially increasing reach and effectiveness. Working more closely with community based organizations could improve trust, particularly as program leadership, reflecting nationwide trends, observed care delays or avoidance due to immigration-related fears (39, 40).

Training adequacy proved another cross-dimensional challenge, affecting program effectiveness, adoption, and implementation. For example, some medical staff, who received a grand rounds-style presentation, were confused by complicated flowcharts and workflows, hindering implementation. Implementation studies in similar settings have found that staff- and site-specific, iterative, ongoing training improved newly-introduced workflow adoption (41) and overall developmental screening rates (22). In addition, staff turnover, which is disproportionately high in community health centers (42), hindered sustained adoption over the project period and threatened program maintenance. Turnover-related strains on staff capacity and institutional memory are well-documented barriers to program adoption in primary care settings (43) and underscore the need for improved retention efforts to ensure new staff can continue implementing ECI without disruption. Finally, as has been shown consistently in existing literature, funding limitations were overwhelmingly cited as primary threats to program sustainability (44).

The experiences of ECI staff indicate a need for top-down, consistent buy-in from all organizational levels as well as each clinic site, particularly for a large multi-site health center network. This is challenging as medical providers are often asked to integrate new screenings and processes into clinical workflows, often without increasing encounter times (45). To begin addressing this challenge, ECI staff met with each new provider to review the program's processes and objectives and distributed detailed program “playbooks.” While this may have increased engagement at the medical provider level, buy-in varied by clinic site and may have been influenced by each site's proportion of pediatric patients, staff knowledge, supervisor support, turnover, or training capacity.

As indicated by the interviewees, organizations that implement programs such as ECI should invest in high quality technology- and workflow-related training for all levels of staff. A technically-proficient staff facilitates the optimization and deployment of digital platforms, benefiting staff and patients alike (22). Digital platforms can offer surveillance and screening tools in multiple languages and formats, opening the door for families who have historically been underserved (46, 47), as well as visual and audio prompts to reduce literacy-related barriers (48, 49). When families understand the purpose of screening and can engage in the process in a culturally and linguistically accessible way, they are more likely to feel connected to their child's developmental journey and more empowered to act on recommendations (46, 50). As suggested by project staff, the larger challenge within the ECI program was ensuring that families access referral information and early intervention services. Technology-enabled systems can send warm referrals directly to early intervention agencies, track follow-up appointment status, and notify care teams when families need support with next steps. This closed-loop referral process guides families rather than leaving them to navigate a complex system on their own. These technology-related benefits, however, are dependent on training frontline staff in their implementation and usage among families. Staff feedback indicates that organizations should also seek to integrate screening and referral workflows into existing EHR systems while ensuring technological solutions allow for easier reporting. Consistent digital screening can potentially support health centers in meeting quality benchmarks tied to state Medicaid programs and value-based payment initiatives (51). Data from these systems help health centers demonstrate strong performance, secure reimbursement, and make the case for continued funding or innovations in early childhood services. In this way, technology-enabled developmental milestone screening is not simply a workflow improvement, but a strategy for advancing equity, strengthening family engagement, and ensuring that children in underserved communities are recognized and supported early, making the greatest impact on lifelong learning, health, and well-being.

This research has several policy implications. First, despite resource limitations, safety net organizations are an ideal setting for innovative child development interventions given their mission-oriented staff and ability to reach underserved families. Technology-enabled developmental milestone screening plays a critical role in strengthening the work of community health centers, allowing teams to identify developmental and socioemotional concerns early, efficiently, and equitably (22). Families served may face challenges such as limited access to pediatric specialists, transportation barriers, language differences, or inconsistent childcare and school engagement, making the well-child visit an ideal opportunity for early detection (52, 53). By embedding screening directly into the clinical workflow, technology ensures that every child is assessed routinely rather than only when a concern is observed. Because effective screening and referral systems offer hefty returns on investment, funders including the Health Resources & Services Administration and Medicaid are recommended to support innovative programs like ECI, as well as relevant technology, in safety net and other settings serving families who face screening barriers. Themes surrounding the difficulties of developmental screening and referrals for children aged three to five years indicate a need for more streamlined policies for children in this age group. In Connecticut, as in many states, services vary by school district. While children between three and five may be offered multiple pathways and support options, these additional decisions can lead to confusion among providers and families alike (54).

While this study has several strengths and key implications for policy, practice, and future research, several limitations are worth noting. While RE-AIM offered a useful organizing framework for the qualitative themes identified, a realist approach may have yielded additional insight into specific contextual factors affecting program implementation. Moreover, while the intervention addresses both developmental screening and service referral, both are complex, interrelated processes that merit separate analyses. The small sample size limits the generalizability of the findings. Moreover, because only 14 out of 20 invitees completed the interview, the results may be subject to self-selection bias. The lack of quantitative data for each RE-AIM dimension prevents a more detailed mixed-methods evaluation. Lastly, while this study focused on providers, it is also important to recognize the need to understand caregiver perception of ECI and similar programs.

Nonetheless, the study's strengths lie in its assessment of staff perception of a novel intervention to enhance early developmental screening and referral, and use of the RE-AIM framework allows stakeholders to examine program elements to guide implementation. The intervention's effectiveness, despite challenges, indicates opportunities for additional research on safety net provider perception of child development surveillance, screening, and referral, as well as the larger scale implementation using lessons learned to improve processes and serve more families.

## Data Availability

The raw data supporting the conclusions of this article will be made available by the authors, without undue reservation.
