# Supplementary material for: Staff perspectives on a novel pediatric developmental screening and referral program in a community health center
Source: Front Pediatr. 2026 Sep 4;14:1872417. doi: 10.3389/fped.2026.1872417 (PMC13585738; doi:10.3389/fped.2026.1872417)
Supplement: Supplementary file 1 [file Supplementaryfile1.docx]

Supplementary Material

# 1 Supplementary Figures and Tables

## 1.1 Supplementary Figures

**Supplementary Figure S1.** ECI workflow for children under age three

**Supplementary Figure S2.** ECI workflow for children aged three to five

**Supplementary Figure S3.** ECI internal dashboard (redacted)

## 1.2 Supplementary Tables

| **RE-AIM DIMENSION** | **NUM.** | **QUESTION** | **PROBE QUESTIONS** |
| --- | --- | --- | --- |
| n/a | n/a: Facilitator opening/ consent | Thank you for joining today’s discussion. My name is [name] and I’m joined by [name] who will be taking notes. We’re both with the Weitzman Institute and are here to learn from your experiences delivering the HRSA-funded Early Childhood Initiative. This is not an evaluation of you or your work—we’re interested in what’s working, what’s challenging, and what can be improved to support better outcomes for children and families. During the discussion, we highly encourage and appreciate concrete examples of what worked or didn’t, which will greatly inform our quality improvement processes.  We also wanted to say that:   - All perspectives are welcome - Speak from your experience - There are no right or wrong answers - We’ll keep your responses confidential (no names used in reporting) - We expect this interview to last no more than 40 minutes.   This meeting will be recorded for our records. No video or audio will be shared with anyone. We will transcribe the material but will de-identify who it is coming from. Only trained research staff will have access to the original recording, but no one else from the program will. Do you consent to recording this interview? | n/a |
| n/a: Warm-up | 1 | Can you briefly share your role and how you’ve been involved with this program? | n/a |
| Reach and Effectiveness | 2 | How well does the program fit with the needs of the children and families you serve? | In your opinion, what are the most essential (core) components of the program? |
|  |  |  | Is the content culturally and linguistically relevant? |
|  |  |  | Any mismatch between what families need and what’s offered? |
|  |  |  | Are there families or groups who aren’t benefiting as much from the program? Who might be left out, and why? |
|  |  |  | How could the program improve appropriateness, access or inclusivity? |
| Adoption and Implementation | 3 | For providers: How manageable has it been to implement the program in your current workflow?  For non-providers: How manageable has it been to support/assist/collaborate with providers in implementing the program? | What has made it easier or harder to deliver? |
|  |  |  | Have time, staffing, or other resources been a challenge? |
|  |  |  | Were the training processes adequate? |
|  | 4 | How supportive is your organization in helping you implement the program? | Is there leadership buy-in? |
|  |  |  | Do you have time and space to implement well? |
|  |  |  | How does this program fit within your other responsibilities? |
|  | 5 | Are you able to deliver the program as designed? | We recognize that adaptation is common. Have you had to make changes or adaptations? If so, what drove those adaptations? To what extent did those changes/adaptations impact outcomes? |
|  |  |  | Do you feel supported in knowing what can be adapted vs. what must remain? |
| Maintenance | 6 | What’s your overall impression of the program? | What do you like most about the program? |
|  |  |  | What aspects feel less useful or relevant to your work? |
|  | 7 | What would it take for this program to continue long-term? | What current supports help? |
|  |  |  | What barriers might threaten sustainability? |
|  |  |  | What keeps you motivated to continue the intervention? |
| n/a: Wrap-up | 8 | What advice would you give to someone new to implementing this program? | n/a |
|  | 9 | Is there anything we haven’t asked that you think is important? | n/a |
| n/a | n/a: Facilitator closing | Thank you for participating today. As we mentioned, the information you provided will only be used to assess the effectiveness and feasibility of the program. At the same time, we are interested in exploring ways to improve its effectiveness and implementation. All information provided will be de-identified, so no one will be able to know what anyone shared. We will be writing a report and can keep you up to date with next steps, including if it is published. For this and any future questions you may have, please do not hesitate to reach out to us.  You can also contact the Principal Investigators [name] or [name]. | n/a |

**Supplementary Table S4.** Semi-structured interview guide

| **RE-AIM DIMENSION** | **DEFINITION^[1]^** | **QUOTE AND RESPONDENT TYPE** |
| --- | --- | --- |
| **REACH** | Were target families served by the ECI program, were gaps recognized, and, if so, how could these gaps be addressed? | “The 3 to 5 age range is the harder target range, because once you finish [the Connecticut] Birth to Three [program], it's not as cut and dry getting services.” *-Clinician* |
|  |  | “We do the best we have as far as a direct translation from the English…but the way that the questions are phrased…may not cross over from the United States-focused approach.” *-Leadership* |
|  |  | “Why might the family not be choosing to move forward? What happened in their family or their past… and what are the stigmas associated with getting support? Being able to start to approach that subject kind of helps to better support that family to get them where you need them to be.”  *-Implementation Staff* |
|  |  | “It sometimes amazes me, how much parents are concerned about their children's development…particularly early in life, and how distracted they are away from that…by things like safety, adequate housing, a multitude of things.” *-Clinician* |
|  |  | “To me, it's just such a critical thing that and having seen some kids where it really made a difference. Where families would have sort of fallen off the…boat.” *-Leadership* |
|  |  | “I think continuing to work with our community partners to advocate that all of our patients get the services that they need continues to be a huge challenge for us.” *-Leadership* |
|  |  | “My hypothesis would be that…we disproportionately miss early diagnosis in non-English speaking patients.” *-Leadership* |
|  |  | “I think concerns about [the Department of Children and Families] and immigration play into some decisions to refuse.” *-Leadership* |
|  |  | “Because we don't offer the evaluations in Spanish…that would be really helpful.” *-Clinician* |
|  |  | “Some families don't think their kids need any sort of interventions…they might grow out of it.” *-Clinician* |
| **EFFECTIVENESS** | Did the program successfully achieve its intended outcomes of early childhood screening and closed-loop follow-ups, and what barriers or facilitators influenced this? | “When we're working together…we were able to…get more families to services and supports.” *-Implementation Staff* |
|  |  | “We're seeing more families accept services and get the support that their child and family may need.” *-Implementation Staff* |
|  |  | “I'm not as…fully well-versed in what is part of the entire program, other than having what we have…at the site right now.” *-Clinician* |
|  |  | “It was a lot of information, and I think we had a hard time… grappling with that flowchart.” *-Clinician* |
| **ADOPTION** | Did the organization successfully apply all aspects of the intervention, which barriers and facilitators influenced program adoption, and were differences observed by role? | “It makes you work harder to make sure these families have what they need.” *-Implementation Staff* |
|  |  | “There is absolutely…leadership buy in.” *-Leadership* |
|  |  | “I don't know if there's, um, like, if this is the highest priority project, right? Um, but I do think, of course, they're supportive and um excited about it.” *-Clinician* |
|  |  | “I remember that they were talking about the way to refer the three-to-five age range. I never managed to integrate that fully into my clinical practice, because I found it very cumbersome and difficult.” *-Clinician* |
|  |  | “That's been the trickiest - just making sure…that all the providers know that we have our process, and that there's a process that they could follow that we've implemented to make their lives easier. So, I think a lot of providers…have their own methods that have worked for them.” *-Implementation Staff* |
|  |  | “Anytime you have turnover…in the provider team…that can create discontinuity for the patient care and the well-child screening process.” *-Leadership* |
|  |  | “After a change in leadership…things fell through the cracks…” *-Clinician* |
| **IMPLEMENTATION** | Was CHCI able to successfully implement the ECI program, what factors made implementation easier or more difficult, and which adaptations were necessary? | “[The care coordinator and community health worker] keep us in the loop…and put out their contact information…so we can easily give it to the families.” *-Clinician* |
|  |  | “Having [program staff] there for me to go up to them and ask them questions…That has made a huge difference.” *-Clinician* |
|  |  | “I mean, our responsibility is really just making sure that those that are developmentally delayed, they're getting the appropriate services and accommodations. So I think if it fits well in terms of that.” *-Clinician* |
|  |  | “[Data] sometimes felt like a barrier, but I think everyone tried to do their best to work together.” *-Support Staff* |
|  |  | “A dashboard was programmed to reel in all the referrals on a daily basis…It’s been great.” *-Implementation Staff* |
|  |  | “[The training] was absolutely adequate…I had the in-person training which was a little over a month.” *-Implementation Staff* |
|  |  | “I think, as part of the onboarding, …it doesn't hurt for folks that have been there a while, new staff, and for everyone to refresh on this.” *-Leadership* |
|  |  | “I think [training] overwhelmed the [medical] staff. They came out just sort of mystified.” *-Leadership* |
|  |  | “The three-to-five [age range] process…was…brand new…so folks definitely needed to adapt…I think that can be very challenging.” *-Clinician* |
|  |  | “We had to make changes…taking the work off providers and taking on more responsibility in the hopes that this would allow providers to refer more children within the three-to-five range.” *-Implementation Staff* |
|  |  | “Every single district has a separate process.”*-Implementation Staff* |
|  |  | “Once kids hit three and transition to the [school system]…we still struggle with that workflow.” *-Leadership* |
| **MAINTENANCE** | What internal or external factors may affect long-term program sustainability or what may need to be modified to ensure this? | “It always comes down to funding, but…we're doing a great job now.” *-Implementation Staff* |
|  |  | “[ECI] is probably underfunded, but… I think a lot of the staff have made up for that with extra work and effort.” *-Clinician* |
|  |  | “Well, I'm unfortunately leaving the organization in a month. So, like, if there's no one who wanted to take my role that would be…it wouldn't destroy the grant, or anything, but it would [not] be ideal.” *-Clinician* |
|  |  | “Use evolving and emerging and developed technology to bypass some of the problems that create that added effort and labor.” *-Clinician* |

**Supplementary Table S5.** RE-AIM dimensions, definitions, and exemplary quotes

1. Adapted from Holtrop JS, Rabin BA, Glasgow RE. Qualitative approaches to use of the RE-AIM framework: Rationale and methods. BMC Health Serv Res. 2018 Mar 13;18(1):177. doi:10.1186/s12913-018-2938-8
